# A region-dependent allele-biased expression of Dopa decarboxylase in mouse brain

**DOI:** 10.3389/fcell.2022.1078927

**Published:** 2022-12-07

**Authors:** Kit-Yeng Sheng, Toru Nakano, Shinpei Yamaguchi

**Affiliations:** ^1^ Department of Pathology, Graduate School of Frontier Biosciences, Osaka, Japan; ^2^ Graduate School of Medicine, Osaka University, Osaka, Japan; ^3^ Stem Cells and Reprogramming Laboratory, Department of Biology, Faculty of Science, Toho University, Chiba, Japan

**Keywords:** imprinting, allelic expression, dopa decarboxylase, brain, reporter mouse, FACS, genomic imprinting, *Ddc*

## Abstract

Genomic imprinting is an epigenetic event in which genes are expressed only from either the paternal or maternal allele. *Dopa decarboxylase (Ddc)*, is an imprinted gene that encodes an enzyme which catalyzes the conversion of L-dopa to dopamine. Although *Ddc* has been reported to be paternally expressed in embryonic and neonatal hearts, its expression pattern in the brain has been controversial. To visualize *Ddc*-expressing neurons, we established a knock-in mouse carrying a humanized Kusabira orange 1 (hKO1) reporter cassette at the *Ddc* locus (Ddc-hKO1). The expression of Ddc-hKO1 was detected in all known *Ddc*-positive cells in the brains of embryonic, neonatal, adult, and aged mice. We further developed an efficient purification method for Ddc-hKO1-positive neurons using a cell sorter. RNA sequencing analysis confirmed the enrichment of dopaminergic, serotonergic and cholinergic neurons in Ddc-hKO1-positive cell population recovered using this method. A detailed analysis of Ddc-hKO1 paternally and maternally derived heterozygous mice combined with immunostaining revealed that *Ddc* was preferentially expressed from the maternal allele in ventral tegmented area (VTA), substantia nigra pars compacta (SNc), and retrorubral field (RRF); while it was expressed from both alleles in dorsal raphe nucleus (DR). These results indicate that *Ddc* exhibit an allele-specific expression pattern in different brain regions, presumably reflecting the diverse regulatory mechanisms of imprinting.

## Introduction

Genomic imprinting is an epigenetic event where the gene expression is geared towards the parent-of-origin manner (Barlow and Bartolomei, 2014). These imprinted genes are crucial in ensuring the proper embryo development and survivability (Kalish et al., 2014; Plasschaert and Bartolomei, 2014; Bonthuis et al., 2015; Prickett et al., 2021). However, the biological significance of imprinted genes in the brain remains unclear due to several technical difficulties, such as anatomically heterogeneity of neuronal and non-neuronal populations, the fragility of neural cells and the extensive networks of synapses which require harsh conditions during the isolation procedures (Poulin et al., 2014; Tiklová et al., 2019). As a result, whole brain or trimmed brain tissue containing heterogenous cell populations has been used as samples for imprinting studies. This approach is only applicable in the case where imprinted genes are uniformly expressed across regions; it is not adaptable when cells with different expression patterns are interspersed. However, previous reports have shown that some imprinted genes in the brain have preferential allelic expression that differs between regions, necessitating a different method of analysis (Gregg et al., 2010).

The monoamine neurotransmitter system in the brain modulates diverse biological functions such as regulating locomotion, learning, neuroendocrine control, and reward (Klein et al., 2019). Abnormalities in dopamine metabolism are closely associated with the development and progression of neuropsychiatric disorders such as Parkinson’s disease, schizophrenia, and developmental disorders (Kesby et al., 2018). Dopamine has important physiological functions not only in the brain but also in the peripheral tissues such as kidneys, pancreas, lungs, and stomach. Dopamine is synthesized from tyrosine in the nervous system by two enzymes. First, tyrosine hydroxylase (TH) catalyzes the conversion of tyrosine to 3,4-dihydroxyphenylalanine (L-DOPA). Then, L-DOPA is converted to dopamine, catalyzes by dopa decarboxylase (*Ddc*). DDC is also known as aromatic L-amino acid decarboxylase (AADC) and it plays an essential role in the dopamine biosynthesis (Lee et al., 2013). *Ddc* deficiency leads to severe autonomic dysfunction and the development of involuntary movements (dystonia), with little to no voluntary movement (Lee et al., 2013).

The expression of a typical imprinted gene is regulated by an imprinting control region (ICR), consists of a differentially methylated region (DMR), which is located in-*cis*, nearby to the imprinting clusters (Edwards & Ferguson-Smith, 2007). *Ddc* is located close to a well-studied imprinting gene, *Grb10*, and shows different patterns of allele-specific expression in different tissues (Menheniott et al., 2008; Gregg et al., 2010; Prickett et al., 2021; Juan et al., 2022). *Ddc*_exon1a, an alternative transcript of *Ddc*, is paternally expressed in trabecular cardiomyocytes of embryonic and neonatal hearts (Menheniott et al., 2008; Juan et al., 2022). The deletions of *Grb10-*DMR of the paternal allele affected the paternally-biased expression of *Ddc* only in the heart (Shiura et al., 2009; Juan et al., 2022). However, its allelic expression preference in the brain was inconsistently reported (Shiura et al., 2009; Gregg et al., 2010; Babak et al., 2015; Bonthuis et al., 2015). Due to its function as a neurotransmitter enzyme in the synthesis of dopamine and serotonin, its expression is not limited to dopaminergic neurons, but also serotonergic and adrenergic neurons, residing in different brain regions (Weihe et al., 2006; Navailles & De Deurwaerdère, 2012). Therefore, understanding the allelic expression profile of *Ddc* in the brain regions is important for elucidating the homeostatic mechanism of neurotransmitter biosynthesis and how its disruption can lead to diseases.

To address these problems, we established a knock-in mouse model bearing a humanized Kusabira-Orange 1 (hKO1) reporter cassette at the *Ddc* gene locus (Ddc-hKO1) to facilitate the detection of *Ddc*-expressing neurons, and developed an efficient purification method for its collection and downstream analyses. Subsequently, we successfully profiled the allelic expression of *Ddc* in five brain regions in the mouse.

## Results

### Establishment of Ddc-hKO1 knock-in reporter mice

**FIGURE 1:**
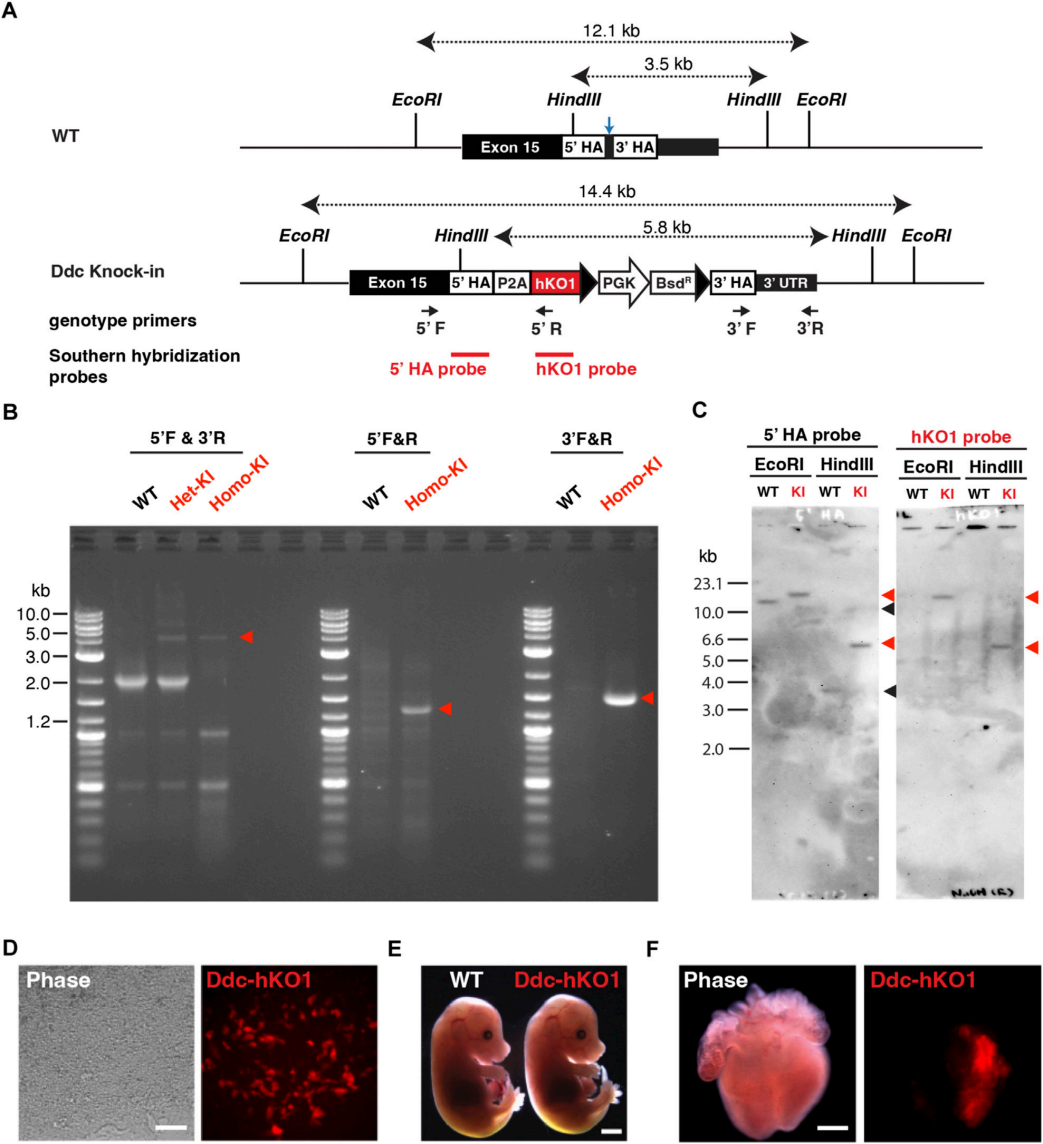
Establishment of Ddc-hKO1 knock-in reporter mice. **(A)** Schematic representation of Ddc-hKO1 knock-in strategy at dopa-decarboxylase (*Ddc*) locus. Arrows indicate the PCR primer sets for the genotyping of the knock-in site. Blue arrow indicates the targeted site of sgRNA. **(B)** Representative results of PCR-based genotyping of wild type, and Ddc-hKO1 knock-in embryonic stem cells (ESC) and mouse. The positions of the primers were indicated in **(A)**. Red arrowheads indicate the expected size of amplicon containing knock-in cassette. DNA ladder size is indicated on the left side of the figure. WT, wild type; Het-KI, Ddc-hKO1 heterozygous ESC; Homo-KI, Ddc-hKO1 homozygous mouse. **(C)** Southern hybridization analysis of Ddc-hKO1 homozygous mouse. Red arrowheads indicate the expected band size of knock-in allele, and black arrowheads indicate the expected band size of wildtype allele. Ladder size is indicated on the left side of the figure. WT, wild type; KI, Ddc-hKO1 homozygous mouse. **(D)** Representative images of cardiac cell lineages differentiated from the knock-in heterozygous ESCs. Scale bar, 50 μm. **(E)** Representative images of E18.5 embryos of WT and Ddc-hKO1 homozygous mice. Scale bar, 2 mm. **(F)** Representative images of E15.5 embryonic heart of Ddc-hKO1 homozygous mouse. Scale bar, 500 μm.

To visualize and purify *Ddc*-expressing neurons, we established a fluorescent reporter knock-in mouse targeting the *Ddc* gene. Using the CRISPR/Cas9-mediated homology-directed repair approach, we inserted a reporter cassette of hKO1 conjugated with a P2A self-cleaving peptide (Figure 1A). After the transfection and clonal expansion of mouse embryonic stem cells (mESCs), successful knock-in was verified using PCR (Figure 1B). Single copy insertion of the knock-in cassette at the *Ddc* gene locus and potential off-target integration was confirmed by Southern blot hybridization analysis (Figure 1C).

*Ddc* is highly expressed in dopaminergic neurons and embryonic cardiomyocytes (Menheniott et al., 2008; Prickett et al., 2021). Therefore, we examined whether the Ddc-hKO1 reporter recapitulated the endogenous expression of *Ddc* using *in vitro* differentiated cardiac cells from knock-in ESCs. As expected, specific expression of hKO1 in spontaneous beating cells was confirmed at 20 days post-differentiation (Figure 1D).

We then established a Ddc-hKO1 knock-in reporter mouse line through chimeric mouse generation using verified knock-in ESCs. No apparent abnormalities in embryonic development or growth were observed in the reporter mouse line (Figure 1E). The adult mice were also healthy and fertile, producing a litter size similar to that of the wild type mice (data not shown). The hKO1 expression was observed in the left atrium and ventricle of the E15.5 embryonic heart, in addition to parts of the brain (Figure 1F and Supplementary Figure S1A). This expression pattern is consistent with the reported *Ddc* expression pattern and indicates the successful insertion of a reporter cassette to the *Ddc* locus (Menheniott et al., 2008; Prickett et al., 2021).

### hKO1 expression recapitulates the endogenous *Ddc* expression in reporter mouse brains

**FIGURE 2:**
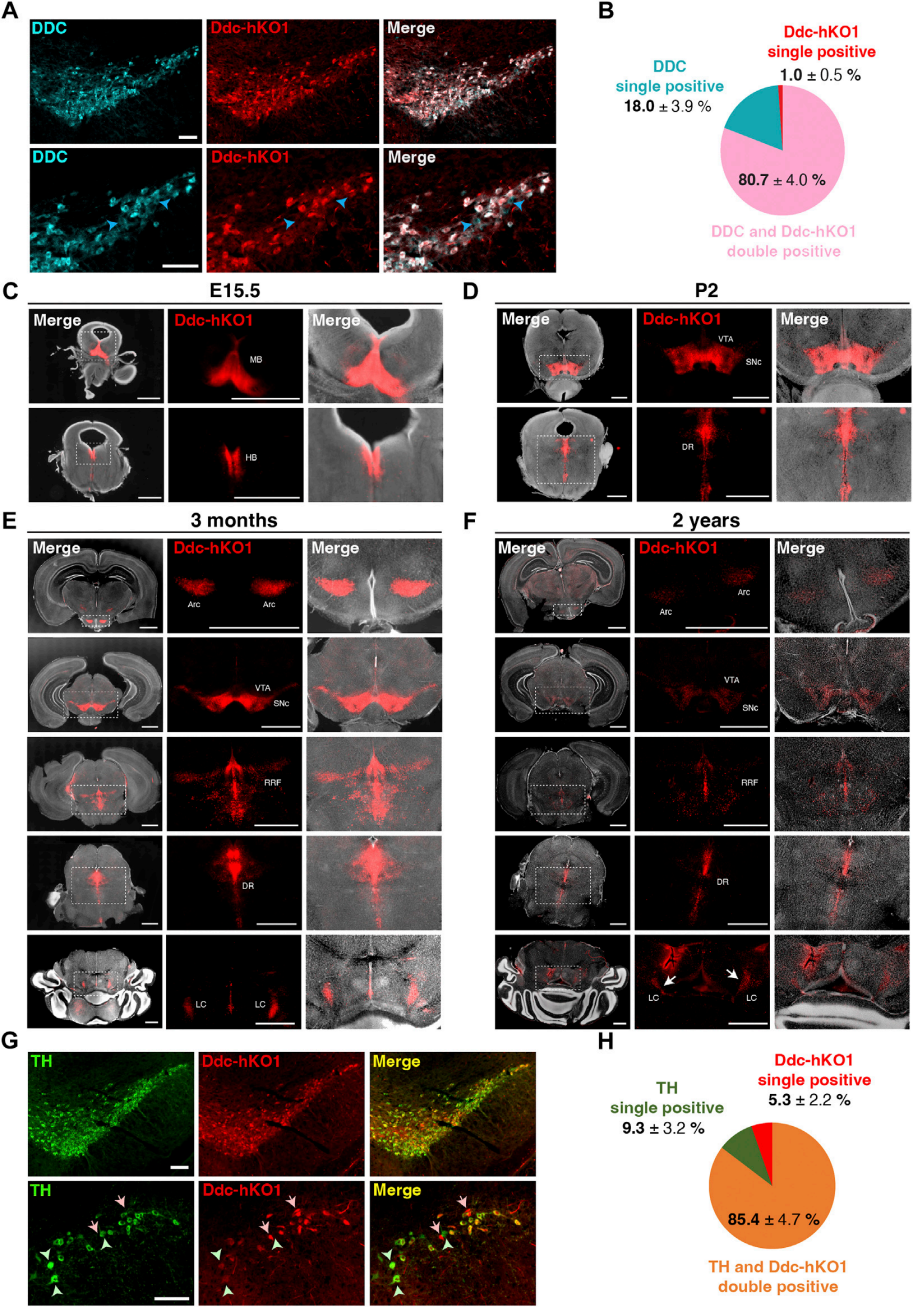
Expression pattern of Ddc-hKO1 in embryonic, neonatal, and adult mouse brains. **(A)** Representative immunostaining images showing the endogenous DDC expression in the VTA and SNc of Ddc-hKO1 homozygous mouse. The enlarged images are shown on the bottom panel. Blue arrowheads indicate DDC-IF single positive cells. Scale bar: 100 μm. **(B)** Pie chart showing the ratio of DDC-IF and Ddc-hKO1-positive populations. ± indicates S.E.M. *n* = 3. **(C–F)** Representative images of vibratome sections of the whole brain in the coronal plane at E15.5 **(C)**, P2 **(D)**, 3 months old **(E)** and 2 years old **(F)** of Ddc-hKO1 homozygous mice. Thickness, 200 μm. Scale bar, 200 μm. MB, midbrain; HB, hindbrain; VTA, ventral tegmental area; SNc, substantia nigra pars compacta; DR, dorsal raphe nucleus; Arc, arcuate nucleus; RRF, retrorubral field; LC, locus coerules. **(G)** Representative immunostaining images showing the TH expression in the VTA and SNc of adult Ddc-hKO1 homozygous mouse, enlarged images are shown on the bottom panel. Arrows indicate Ddc-hKO1 single positive cells, green arrowheads indicate TH single positive cells. Scale bar: 100 μm. **(H)** Pie charts showing the ratio of TH and Ddc-hKO1 positive populations. ± indicates S.E.M. *n* = 4.

To determine whether Ddc-hKO1 expression reflects endogenous *Ddc* expression, we performed immunostaining of the VTA and SNc region of adult mouse brain using a DDC antibody (Figure 2A). The DDC immunostaining (DDC-IF) signals were found to colocalize with more than 80% of the Ddc-hKO1-singals (Figure 2B). About 18% of DDC-IF-positive cells were negative for Ddc-hKO1. Importantly, only 1% of Ddc-hKO1-positive cells were not DDC-IF-positive (Figure 2B). These results can be explained by the necessity of antigen retrieval procedure for DDC staining, which potentially affects the hKO1 signal. Next, to strengthen our observation, we evaluated the expression pattern of the Ddc-hKO1 reporter in four developmental stages: prenatal (E15.5), neonatal (2 days after birth, P2), young adult (3 months old), and aged mice (2 years old). At the prenatal stage, Ddc-hKO1-positive cells resided in the midbrain and hindbrain regions (Figure 2C and Supplementary Figure S1A). Similarly, Ddc-hKO1-positive cells were observed in the VTA and SNc in the midbrain and the dorsal raphe nucleus (DR) in the hindbrain of newborn pups and adult mice (Figures 2D,E, Supplementary Figures S1B,C). We also detected Ddc-hKO1-positive cells in the arcuate nucleus (Arc) in the hypothalamus, retrorubral field (RRF), and locus coeruleus (LC) in the hindbrain of an adult mouse brain (Figure 2E, Supplementary Figure S1C).

An expression pattern of Ddc-hKO1 was similarly observed in aged mice, but its intensity was weaker in most regions (Figure 2F). Overall, the expression patterns of Ddc-hKO1 in the brain are virtually identical to the results of *in situ* hybridization of *Ddc* in the Allen Mouse Brain Atlas (www.brain-map.org) (Lein et al., 2007) and the Allen Developing Mouse Brain Atlas (http://developingmouse.brain-map.org). Additionally, no Ddc-hKO1-positive cells were found in other brain regions, which was in good agreement with the reported *Ddc* expression pattern. This expression pattern is consistently observed in different generations of reporter mice. Next, we investigated the expression pattern of TH in Ddc-hKO1-positive cells in the midbrain. Immunostaining of the VTA and SNc regions revealed that more than 85% of the Ddc-hKO1-positive cells expressed high levels of TH (Figures 2G,H). On the other hand, 9.3% and 5.3% of TH and Ddc-hKO1 single-positive cells were also observed, respectively. This result is consistent with a previous study reporting the presence of subpopulations of DA neurons with differential expression of *Ddc* and *Th* (Weihe et al., 2006). Altogether, these observations further corroborate that the reporter expression reflected the endogenous *Ddc* expression.

### Purification of *Ddc*-expressing neurons using Ddc-hKO1 reporter mice

**FIGURE 3:**
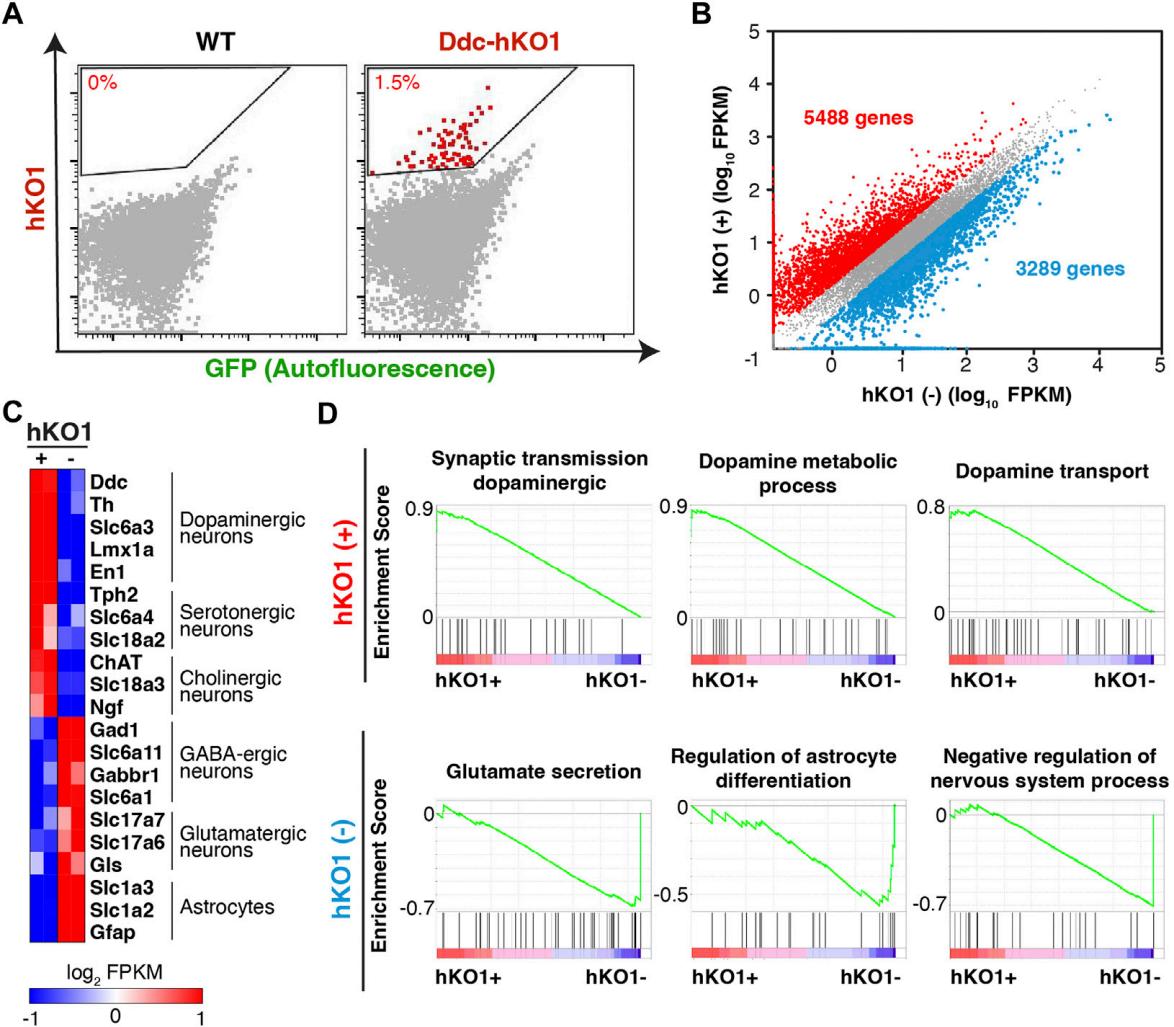
Purification of dopaminergic neurons from Ddc-hKO1 homozygous mouse. **(A)** Representative FACS dot plots showing the gating strategy for the recovery of hKO1-positive neurons from Arc, VTA, SNc, DR, RRF, and LC of WT and Ddc-hKO1 homozygous mouse brains. Red dots represent the hKO1-positive neurons. **(B)** Scatter plot of RNA-Seq gene expression data comparing hKO1-positive and hKO1-negative populations. Fold change (FC) > 2. Red dots indicate genes highly expressed in hKO1-positive neurons and blue dots indicate genes highly expressed in hKO1-negative cells. **(C)** Heat map representation of expression levels of selected neuronal and non-neuronal marker genes. **(D)** Representative gene set enrichment analysis (GSEA) results enriched in hKO1-positive and hKO1-negative populations.

A reporter mouse expressing a fluorescent protein offers the advantage of allowing the purification of fluorescence positive cells using a Fluorescence-activated cell sorter (FACS). We next asked whether Ddc-hKO1 reporter mice could enrich *Ddc-*expressing neurons using FACS for subsequent analyses such as RNA sequencing (RNA-seq). The regions containing hKO1-positive cells in midbrains of adult mice were micro-dissected and subjected to enzymatic digestion to obtain a single-cell suspension (Supplementary Figure S2A). Pre-equilibration of the media with 95% oxygen and supplementation of D-(+)-trehalose before and during the dissociation process were two key factors that greatly enhance the survivability of neurons (Saxena et al., 2012). We successfully collected an average of 14,000 viable hKO1-positive neurons with the detection of red fluorescence signals (approximately 1.5%) from each brain (Figure 3A, Supplementary Figure S1B). We pooled two adult brains as one biological replicate for the following analyses to ensure sufficient yield and to minimize variation among samples.

To verify that the purified hKO1-positive cells are viable *Ddc*-expressing neurons, we analyzed their transcriptome by RNA-seq. Among the 24,346 mapped genes, 5,488 and 3,289 genes showed significantly higher expression in the hKO1-positive and hKO1-negative cells, respectively (fold change [FC] > 2) (Figure 3B). The typical marker genes for dopaminergic (*Ddc*, *Th*, *Slc6a3*, *Lmx1a*, and *En1*), serotonergic (*Tph2*, *Slc6a4*, and *Slc18a2*), and cholinergic neurons (*ChAT*, *Slc18a3*, and *Ngf*) were highly expressed in the hKO1-positive population but not in the Ddc-hKO1-negative population (Figure 3C). In contrast, a group of genes that should be highly expressed in *Ddc*-negative populations, such as GABAergic (*Slc6a11*, *Gabbr1*, and *Slc6a1*), glutamatergic neurons (*Slc17a6*, *Slc17a7*, and *Gls*), and astrocytes (*Slc1a2*, *Slc1a3*, and *Gfap*), were highly expressed in the Ddc-hKO1-negative population (Figure 3C). In addition, we performed gene set enrichment analysis (GSEA) on all mapped genes to gain insights into the biological processes in both hKO1-positive and -negative populations. Biological processes, such as synaptic transmission in the dopaminergic neuron process (normalized enrichment score [NES] = 1.92), dopamine metabolic process (NES = 1.88), and dopamine transport (NES = 1.78), appeared on the top of the list of positively correlated enriched gene sets (Figure 3D). Gene sets related to catecholamine biosynthesis (NES = 1.95) and catecholamine metabolic processes (NES = 1.94) were also positively enriched. Meanwhile, biological processes, such as regulation of glutamate secretion (NES = −0.80), regulation of astrocyte differentiation (NES = −0.57), and negative regulation of the nervous system process (NES = −0.71), were negatively enriched (Figure 3D). These results confirm that the sorted hKO1-positive population is indeed comprises *Ddc*-expressing neurons.

### Brain region-specific allelic-biased expression of *Ddc*

**FIGURE 4:**
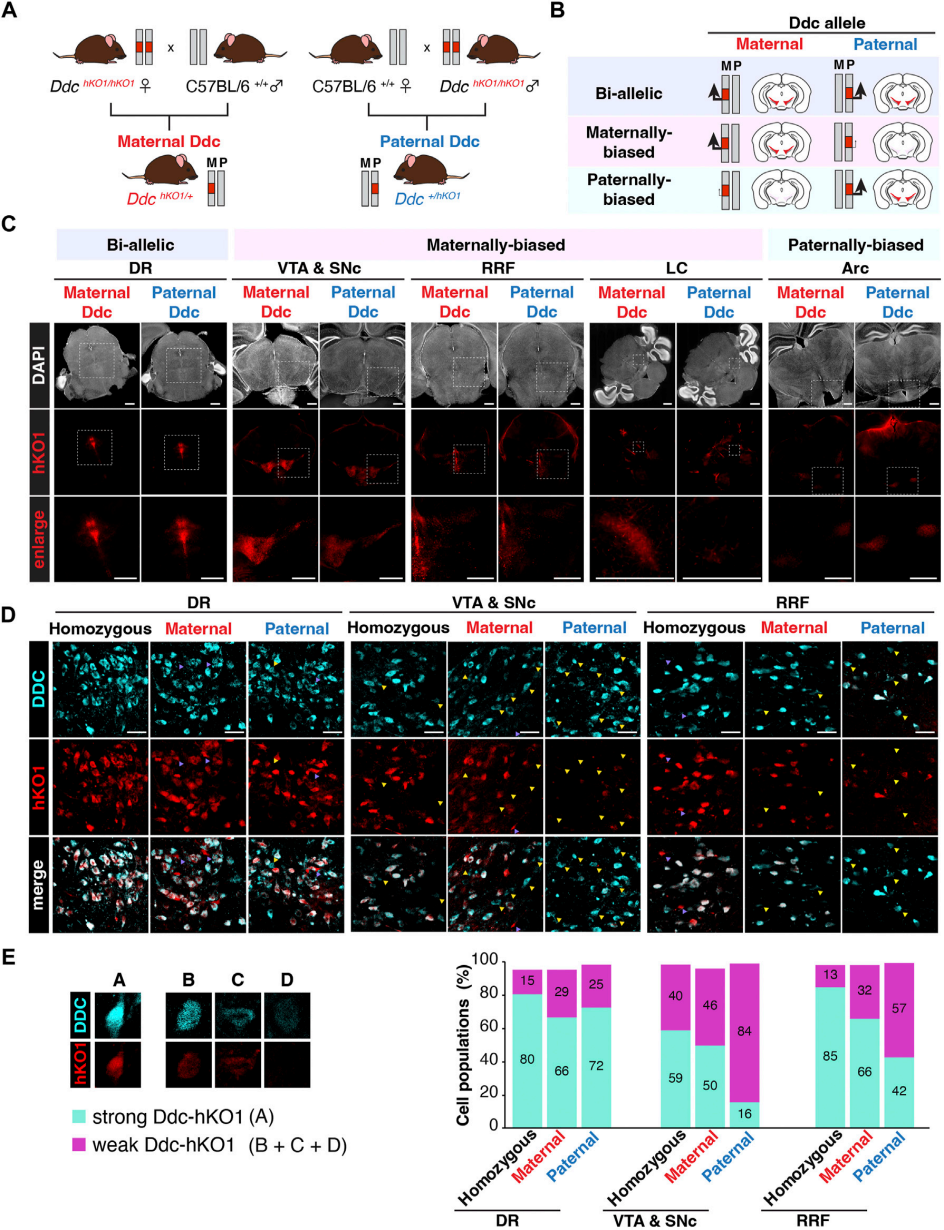
Allelic expression of *Ddc* in the mouse brain. **(A)** Illustration showing the crosses of Ddc-hKO1 homozygous mouse with wild type mouse to obtain single allele with hKO1 knock-in, either inherited maternally (maternal *Ddc*) or paternally (paternal *Ddc*) in heterozygous mice for the investigation of the allelic expression of *Ddc* in the brain. **(B)** Illustration showing the expected hKO1 expression and fluorescence intensity signal for each combination of allelic expression pattern in maternal and paternal *Ddc*. **(C)** Representative images showing hKO1 expression in *Ddc*-positive mouse brain regions of maternal and paternal *Ddc*. Scale bar, 1,000 μm) **(D)** Representative images showing DDC immunofluorescence (DDC-IF) and Ddc-hKO1 reporter expression in DR, VTA and SNc, and RRF regions of Ddc-hKO1 homozygous, maternally derived and paternally derived heterozygous mice. Yellow triangle indicates single positive of IHC, purple triangle indicates single positive of hKO1. *n* = 3; Scale bar, 50 μm. **(E)** Bar graph showing the percentage of cell numbers of DDC-IF and Ddc-hKO1 positive populations. Double positive cells are categorized as (A) and Ddc-hKO1-weak or -negative cells are categorized as (B+C+D), according to the DDC-IF intensity, as represented in the legend on the left panel. *p < 0.05, **p < 0.001, *t*-test, *n* = 3. DR, dorsal raphe nucleus; VTA, ventral tegmental area; SNc, substantia nigra pars compacta; LC, locus coeruleus; Arc, arcuate nucleus; RRF, retrorubral field.

Since the allele-specific expression profile of *Ddc* in the mouse brain was contradictory and inconclusive in several studies, we decided to investigate the allelic expression pattern of *Ddc* in various brain regions using Ddc-hKO1 mice. Female and male Ddc-hKO1 homozygous mice were crossed with wild type mice to obtain heterozygous mice that inherits the Ddc-hKO1 reporter allele maternally or paternally (Figure 4A). We analyzed the *Ddc* expression bias of maternal and paternal alleles by comparing the Ddc-hKO1 signal intensity in maternally and paternally derived heterozygous mice (Figure 4B). Microscopy observation of cleared vibratome sections of five brain regions revealed that Ddc-hKO1 is expressed from both maternal and paternal alleles in all regions, but the expression bias varies with regions (Figures 4C,D). The signal intensities of Ddc-hKO1 in the DR of maternally and paternally derived heterozygous mice were similar, indicating a bi-allelic expression of *Ddc* (Figure 4C). Meanwhile, the brain regions, such as the VTA and SNc, RRF, and LC, displayed stronger Ddc-hKO1 fluorescence signal in the maternal *Ddc* compared to the paternal *Ddc* (Figure 4C). In contrast, we observed a stronger hKO1 fluorescence signal in the Arc in the hypothalamus region of the paternally derived Ddc-hKO1 heterozygous mouse brain, suggesting that *Ddc* was expressed in Arc in a paternally-biased manner (Figure 4C).

Vibratome sections can be used to analyze the expression trends of allele-specific bias in a broad region. However, since sections from different mice were analyzed, the number of *Ddc*-positive cells may vary in the observed areas. Therefore, we performed immunostaining using a DDC antibody to confirm that the difference in hKO1 expression was indeed due to biased allelic expression. The DDC-IF represents the total expression of DDC from both alleles, while the Ddc-hKO1 signal represents the expression of single allele (Figure 4D). The DDC-IF-positive/Ddc-hKO1-negative cells are therefore thought to express *Ddc* monoallelically (B + C + D, from Figure 4E and Supplementary Figure S3A). It will be possible to identify maternally biased expression by the presence of Ddc-hKO1 signals in the majority of DDC-IF-positive cells of maternally derived Ddc-hKO1 heterozygous mice (A, Figure 4E, Supplementary Figure S3A), whereas Ddc-hKO1 signals can only be detected to a lesser extent in paternally derived Ddc-hKO1 heterozygous mice (B + C + D, Figure 4E and Supplementary Figure S3A). In the case of paternally-biased expression, the opposite observation would be expected. If *Ddc* is biallelically expressed, then Ddc-hKO1 signals will be detected in both maternally and paternally derived Ddc-hKO1 heterozygous mice.

To evaluate the effects of the heat antigen retrieval process on immunostaining and hKO1 signals, Ddc-hKO1 homozygous mice were included as a control. Even in Ddc-hKO1 homozygous mice, 0.5%–4.9% of cells were Ddc-hKO1-positive but DDC-IF-negative, which would be due to technical reasons such as antibody accessibility (Supplementary Figures S3A–D). About 70% of DDC-IF-positive cells in both maternally and paternally derived heterozygous mice were Ddc-hKO1-positive in DR, suggesting that both paternal and maternal alleles were expressed at the same level (Figures 4D,E, Supplementary Figures S1A,B). On the other hand, the percentage of Ddc-hKO1-positive cells in RRF was lower in paternally derived heterozygous mice than in maternally derived heterozygous mice (66% vs 42%) (Figures 4D,E, Supplementary Figures S1A,D, and this tendency was more pronounced in VTA and SNc (50% vs. 16%) (Figures 4D,E, Supplementary Figures S1A,C). These results are consistent with the stronger signal observed in the vibratome sections of Ddc-hKO1 maternally derived heterozygous mice (Figure 4C), indicating that *Ddc* is preferentially expressed from maternal alleles in these regions. Due to the technical difficulties of immunostaining with DDC antibodies, this method was not suitable for LC and Arc.

## Discussion

Since the discovery of imprinted genes, much interest has been dedicated to understanding their biological importance and regulatory mechanism. Concurrently, multiple efforts were made in attempting to bulk screen for more imprinted genes in mice, and it had been mainly relied on sequencing analysis. An allelic-biased expression was usually profiled using hybrid mice bearing SNPs between maternal and paternal alleles. However, this approach has a couple of technical limitations. First, only a small number of genes harbor a reliable number of SNPs in their gene bodies. Second, strain-specific expression bias should be assessed by the reciprocal crossing, as it is more pronounced than allele-specific expression. Additionally, conventional sequencing-based approaches could mask the allele-specific gene expression in certain cell populations, since tissues generally consist of different cell types and subpopulations. The establishment of the reporter mouse provides an alternative method for imprinting studies as it allows straightforward analysis by observing fluorescent reporter signals as a representation of gene transcription (Garfield et al., 2011; Bonthuis et al., 2015). Therefore, our reporter mouse is particularly useful in studying *Ddc* in the brain since it allows us to overcome the complexity of the population heterogeneity.

Our reporter mouse revealed that *Ddc* was expressed in a paternally-biased manner in the Arc, in a maternally-biased manner in the VTA and SNc, RRF, and LC, and in a biallelic manner in DR. Very recently, a similar approach reported a maternal allele preferential expression of *Ddc* in the mouse brain to a different extent in different regions (Bonthuis et al., 2015). Their results and ours were mostly consistent, but we obtained a different result showing paternally-biased expression in Arc. We reasoned that this could be due to different fluorescent proteins and detection approaches were used. We analyzed brain vibratome sections of maternally and paternally derived heterozygous mice while they utilized dual eGFP and G5-tag-knock-in mice, which might cause a difference in detection sensitivity of *Ddc* at the Arc. One of the key open questions is the underlying molecular mechanisms regulating this region-specific, allele-specific expression. Deletion of the Grb10-DMR had a negligible effect on *Ddc* expression in the whole embryo, yolk sac, or liver, but its expression in brain subtypes has not been analyzed (Shiura et al., 2009). Analyses of Ddc-hKO1 mice with Grb10-DMR deletion would allow us to address this point. On the other hand, how regions within the brain give rise to distinct cellular characteristics is mostly unknown. Combining the use of reporter mice with single-cell transcriptomic analysis may be a possible approach in the future.

We also demonstrated that the *Ddc* expressing population could be enriched with the optimized neural cell isolation protocol for downstream analyses such as RNA-seq. It would be applicable to the study of dopaminergic neuron subtypes which show distinct expression patterns in marker genes (Weihe et al., 2006; Poulin et al., 2014; Tiklová et al., 2019; Fernandes et al., 2020). Although scRNA-seq and immunohistochemical analyses have demonstrated the heterogeneity of dopaminergic subpopulations in the brain, their specific function remained elusive. Different susceptibilities toward toxin treatment were previously observed among the subpopulations of dopaminergic neurons (Poulin et al., 2014). The dual reporter mouse systems, such as Ddc-hKO1/Th-GFP, would reveal the nature and function of such subpopulations. Investigating the physiological function of these subpopulations may offer valuable insights into selective vulnerability in degeneration and drug development.

In summary, we revealed that *Ddc* imprinted expression is region-specific in the mouse brain. Our report provides the *Ddc* expression profile in the mouse brain, which serve as a base reference platform for studying brain functions. Additionally, our Ddc-hKO1 reporter mice offer an efficient purification system of neurons with high quality and quantity of yield for downstream analyses, which could be extensively exploited for various molecular pathways and biological function of *Ddc* in brain development and maintenance.

## Materials and methods

### Animals

All animal experiments were conducted with approval and in compliance with the guidelines of the Animal Care and Use Committee of the Graduate School of Frontier Biosciences, Osaka University. All studies were carried out in compliance with the ARRIVE guidelines. Mice were kept under standard laboratory conditions with controlled temperature and 12 h light/dark cycle with *ad libitum* access to food and water intake. All mice used in this study, including ICR and C57BL/6J, were purchased from Japan SLC (Hamamatsu, Japan). The day the vaginal plug was observed was defined as embryonic day 0.5 (E0.5).

### Vector construction for Ddc-hKO1 knock-in

Knock-in of the hKO1 cassette into the *Ddc* locus was conducted using CRISPR/Cas9-mediated homology-directed repair genome editing. The construction design of the Ddc-hKO1 donor vector is shown in Figure 1A. The 5′ homologous arm (HA), which is 732 bp upstream before the stop codon, and 3′ HA, which is 602 bp downstream after the stop codon, were amplified from mouse genomic DNA using the following primer sets: 5′ HA: forward, 5′-TCG​AAT​TCG​CGG​ATC​CTT​AGT​CAT​TGG​GAG​TGG​AG-3′; reverse, 5′-TAG​TAG​CTC​CGG​ATC​CTT​CTT​TCT​CTG​CCC​TCA​GC-3′; 3′ HA: forward, 5′-ACG​AAG​TTA​TCT​TAA​GAG​GCA​TCA​GGA​TTC​CAG​C-3′, reverse, 5′-CGG​TGG​CGG​CCT​TAA​GAG​CTG​GCA​ATG​TAG​CTC​AG-3′. The single guide RNA target sequence of the insertion site was 5′-CAG​GTA​AGC​TAG​CTG​CAC​CA-3′.

### Cell culture and transfection

A G4 mouse embryonic stem cell (ESC) line was used in this study (George et al., 2007). ESCs were maintained under serum-free conditions [0.5 × N-2 (Thermo Fisher Scientific, MA, United States), 0.5 × B-27 (Thermo Fisher Scientific, MA, United States), 100 U/ml mouse LIF (Merck, NJ, United States), 3 μM CHIR99021 (Funakoshi, Tokyo, Japan), 1 μM PD0325901 (Stemgent, MA, United States), 1 mM L-glutamine (Nacalai Tesque, Kyoto, Japan), and 1 × penicillin/streptomycin (Invitrogen, MA, United States) in DMEM/F-12 with GlutaMAX (Thermo Fisher Scientific, MA, United States)] in the absence of feeder layers. The donor vector and pX330 Cas9/single guide RNA vector were transfected into ESCs using Lipofectamine 2000 (Thermo Fisher Scientific, MA, United States). Successful knock-in of the reporter cassette was confirmed by PCR amplification. Primer sets for 5′ and 3′ insertion sites were as follows: forward, 5′-TGA​AGC​CTG​AAA​CCA​GCC​CC-3′, reverse, 5′-GCT​CGA​AGC​AGT​TGC​CCC​TCA-3′; and forward, 5′-ACG​GCA​GTT​GGG​ATT​CGT​GA-3′, reverse, 5′-CAT​GAT​GAC​CAA​GTG​TCT​GAA​AGG​G-3′, respectively. The expected PCR product size for wild type is 2,132 bp (5′ forward and 3′ reverse); for knock-in are 4,440 bp (5′ forward and 3′ reverse), 1,357 bp (5′ forward and 5′ reverse) and 1,574 bp (3′ forward and 3’ reverse).

### Cardiac lineage differentiation of ESCs

Cardiac cell differentiation using Ddc-hKO1 ESCs was performed as previously described (Pucéat, 2008). Briefly, ESCs were treated with 2.5 ng/ml of BMP-2 (Bio-Techne, Minnesota, United States) in propagation medium [100 mM non-essential amino acids (Thermo Fisher Scientific, MA, United States), 100 U/ml mouse LIF, 20 µM β-mercaptoethanol (Nacalai Tesque, Kyoto, Japan), 1 × penicillin/streptomycin (Invitrogen, MA, United States of America), and 10% FBS [Nichirei, Tokyo, Japan] in DMEM/F-12 with GlutaMAX] for 24 h. ESCs were then passaged for embryoid body formation using the hanging drop method for 3 days. Embryoid bodies were collected in differentiation medium (100 mM non-essential amino acids, 20 μM β-mercaptoethanol, 1 × penicillin/streptomycin (Invitrogen, MA, United States), and 20% FBS in DMEM/F-12 with GlutaMAX) and transferred to an ultra-low adhesion plate (Wako, Osaka, and Japan) for 3 days to allow further differentiation under floating culture conditions. Embryoid bodies were plated on a cell culture dish coated with 0.1% gelatin (Sigma-Aldrich, Missouri, United States), and spontaneous beating cells were observed at approximately 7 days after attachment.

### Establishment of reporter mice

Ddc-hKO1 chimeric mice were generated using the aggregation method. Briefly, 8–16-cells stage embryos were collected from the oviduct ampulla and uterus by flushing with M2 medium (Sigma-Aldrich, Missouri, United States) at E2.5. Zona pellucida was digested using 0.5% pronase (Sigma-Aldrich, Missouri, United States), and each morula was aggregated with Ddc-hKO1 ESCs. Following overnight incubation, chimeric blastocysts were transferred into the uterine of pseudo-pregnant ICR female mice. Chimeric mice were crossed with C57BL/6 female mice for several generations to maintain their C57BL/6J background. The primer sets for genotyping were as follows: forward, 5′-GTT​TGT​GCT​ACG​CTT​TGC​TG-3′; reverse, 5′-CCT​CAG​GGT​CAT​CTC​CTG​GT-3′.

### Southern hybridization

Genomic DNA collected from brains of C57BL/6J and Ddc-hKO1 homozygous mice was digested with *EcoR*I and *Hind*III overnight. Digested DNA (2 mg) was separated in an agarose gel and blotted onto the nylon membrane (Amersham Hybond-N; GE Healthcare, United Kingdom). Probes were labeled using PCR-DIG probe synthesis kit (Roche, Basel, Switzerland). Probes were detected with anti-Digoxigenin-AP fragments (Roche, Basel, Switzerland) and CDP-Star solution (Roche, Basel, Switzerland). The probe sequence for 5′HA and hKO1 were as follows: 5′HA forward, 5′-TCG​AAT​TCG​CGG​ATC​CTT​AGT​CAT​TGG​GAG​TGG​AG-3′; 5′ HA reverse, 5′-TAG​TAG​CTC​CGG​ATC​CTT​CTT​TCT​CTG​CCC​TCA​GC-3′; hKO1 forward: 5′-CGA​GGA​GAT​CCC​CGA​CTA​CT-3′, hKO1 reverse, 5′-GGC​AAA​CAA​CAG​ATG​GCT​GG-3′.

### Vibratome sectioning and imaging

Brains were collected from Ddc-hKO1 homozygous mice and fixed in 4% paraformaldehyde in phosphate buffered saline (4% PFA/PBS) overnight at 4°C. Fixed brains were embedded in freshly prepared 3% low-melting temperature agar before sectioning. Embedded brains were sectioned using a vibratome (Leica VT1000s; Leica Microsystems, Wetzlar, Germany) with a thickness of 200 μm and placed in chilled PBS. Sections were treated with 0.5× CUBIC-1, an animal tissue clearing reagent, for 24 h, followed by 1× CUBIC-1 for another 24 h (Susaki et al., 2014). Sections were mounted in 1× CUBIC-1 with 4′,6-diamidino-2-phenylindole, and imaged using a fluorescence microscope (BZ-X700; Keyence, Osaka, Japan).

### Brain dissociation and cell sorting using a FACS

The brain dissociation protocol was performed with modification as previously described (Fischer et al., 2011; Walker & Kempermann, 2014). Ddc-hKO1 homozygous mouse brains were harvested and placed in chilled 95% oxygenated hibernate A solution (Thermo Fisher Scientific, MA, United States). The VTA and SNc of the midbrain were micro-dissected in chilled dissection medium (20 mM HEPES [Nacalai Tesque, Kyoto, Japan], 10% [w/v] D-(+)-trehalose dihydrate [Nacalai Tesque, Kyoto, Japan] in HBSS (+) with phenol red [Nacalai Tesque, Kyoto, Japan]) saturated with 95% oxygen. The recovered VTA and SNc were minced into smaller blocks covered with dissection medium and immediately transferred into pre-warmed dissociation medium (10 U papain [Worthington, Ohio, United States], 2 mg DNase I [Sigma-Aldrich, Missouri, United States], and 4 U Dispase II [Sigma-Aldrich, Missouri, United States] in HBSS (+) with phenol red). After a 30-min incubation at 37°C, tissues were gently dissociated using a fire-polished glass pipette (approximately 40 μm of internal diameter). Brain suspension was resuspended with solution A (20 mM HEPES, 40 mg/ml BSA [Nacalai Tesque, Kyoto, Japan], 10% [w/v] D-(+)-trehalose dihydrate, 4 μl of RNase inhibitor [Nacalai Tesque, Kyoto, Japan] in HBSS without phenol red), followed by filtration using a cell strainer (pore size 40 μm). The filtrate was subjected to centrifugation, and the collected pellet was resuspended in solution B [0.9 M sucrose (Nacalai Tesque, Kyoto, Japan), 10% (w/v) D-(+)-trehalose dihydrate, 4 μl of Rnase inhibitor in HBSS without phenol red, pH 7.5]. Neuronal cells were concentrated by centrifugation in solution B. Finally, the cell pellet was washed again with solution A and resuspended in sorting medium [2% FBS (Thermo Fisher Scientific, MA, United States), 10% [w/v] D-(+)-trehalose dihydrate, 40 U Rnase inhibitor (Thermo Fisher Scientific, MA, United States) in DMEM/F-12 without phenol red [Thermo Fisher Scientific, MA, United States)]. All procedures were conducted on ice except for the digestion process. The hKO1-positive and -negative cells were sorted using a FACS (BD FACS Aria III, BD Bioscience, United States) with 100 μm flow cells at a flow rate of 1.

### RNA purification and RNA-seq analysis

hKO1-positive and -negative cells were collected by sorting into TRIzol LS reagent (Thermo Fisher Scientific, MA, United States). Cells from the two brains were pooled as one biological replicate. RNA purification was performed using a Direct-zol RNA microprep kit (Zymo Research, CA, United States) according to the manufacturer’s protocol. Library preparation was performed using the SMARTer Ultra-Low RNA Kit (Clontech, CA, United States), and cDNA was amplified according to the manufacturer’s protocol. Sequencing was conducted using a next-generation sequencer, an Illumina NovaSeq 6,000 platform in 101-base single-end mode. Sequence reads were mapped to mouse reference genome sequences (mm10) using TopHat software (v 2.0.13) combined with Bowtie2 (v 2.2.3) and SAM tools (v 0.1.19). The FPKM values were calculated using Cufflinks software (v 2.2.1). Gene ontology enrichment analysis was performed using DAVID functional annotation bioinformatics microarray analysis (https://david.ncifcrf.gov) and ranked GSEA (v 4.1.0) (http://www.gsea-msigdb.org/gsea/index.jsp).

### Immunohistochemistry

Adult male mice (two to 3 months old) were deeply anesthetized and perfused transcardially with 4% PFA/PBS. Dissected brains were post-fixed overnight in 4% PFA/PBS and then immersed in PBS containing 30% sucrose solution (30% sucrose/PBS) until sinking as reported previously (Furuya et al., 2004). Immunohistochemistry was performed on 20 μm serial sections, sectioned with a cryostat (Leica Microsystems, Wetzlar, Germany). The primary antibodies used were as follows: rabbit anti-DDC ab3905 (1:1,000, Abcam, Cambridge, United Kingdom) and rabbit anti-TH (1:1,000; Calbiochem, CA, United States). For double immunofluorescence staining, appropriate fluorescent secondary antibodies conjugated to Alexa Fluor 647 (1:500; Abcam, Cambridge, United Kingdom) were used. Incubation was performed in PBS for 1 h at room temperature. Sections were washed with PBS three times, counterstained with 4′,6-diamidino-2-phenylindole mounting medium (Vectashield, Vector Laboratories), and observed using BZ-9000 (Keyence, Osaka, Japan) or LSM800 with AiryScan (Zeiss, Jena, German). The immunofluorescence of DDC was analyzed in three biological replicates, and three cryosections of the VTA and SNc regions were analyzed for each mouse as technical replicates. A total of 100–300 DDC-IF-positive cells were analyzed in each section. For the stereological assessment of the total number of TH-positive neurons, serial sections were prepared as reported previously (Furuya et al., 2004). Every fourth section was stained through the entire extent of the SNc. Cells were counted based on the method described by Furuya et al. (2004).

## Data Availability

The datasets presented in this study can be found in online repositories. The names of the repository/repositories and accession number(s) can be found below: All RNA-seq datasets have been deposited in the NCBI Gene Expression Omnibus (GEO) with the accession number GSE171581.
