# Supplementary material for: A region-dependent allele-biased expression of Dopa decarboxylase in mouse brain
Source: Front Cell Dev Biol. 2022 Dec 7;10:1078927. doi: 10.3389/fcell.2022.1078927 (PMC9768605; doi:10.3389/fcell.2022.1078927)
Supplement: Supplementary file 1 [file DataSheet1.PDF]

Supplementary Figure 1

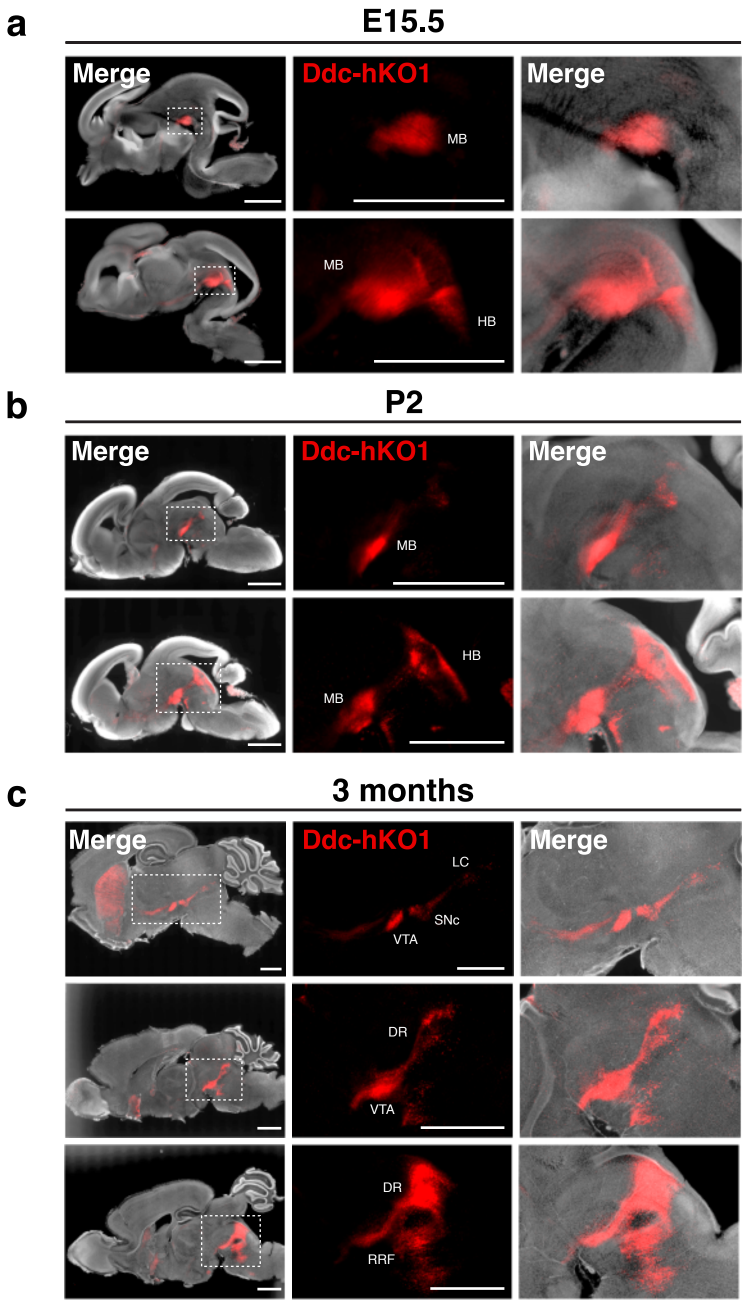

Supplementary Figure 1

Characterization of Ddc-hKO1 expression in embryonic, neonatal, and adult brains. **(a-c)** Representative images of vibratome sections of whole brain of Ddc-hKO1 homozygous mice in the sagittal plane at E15.5 **(a)**, P2 (**b**), and 3 months old **(c)**. Thickness, 200 µm; Scale bar, 200 µm. MB, midbrain; HB, hindbrain; VTA, ventral tegmental area; SNc, substantia nigra pars compacta; DR, dorsal raphe nucleus; RRF, retrorubral field; LC, locus coeruleus.

Supplementary Figure 2

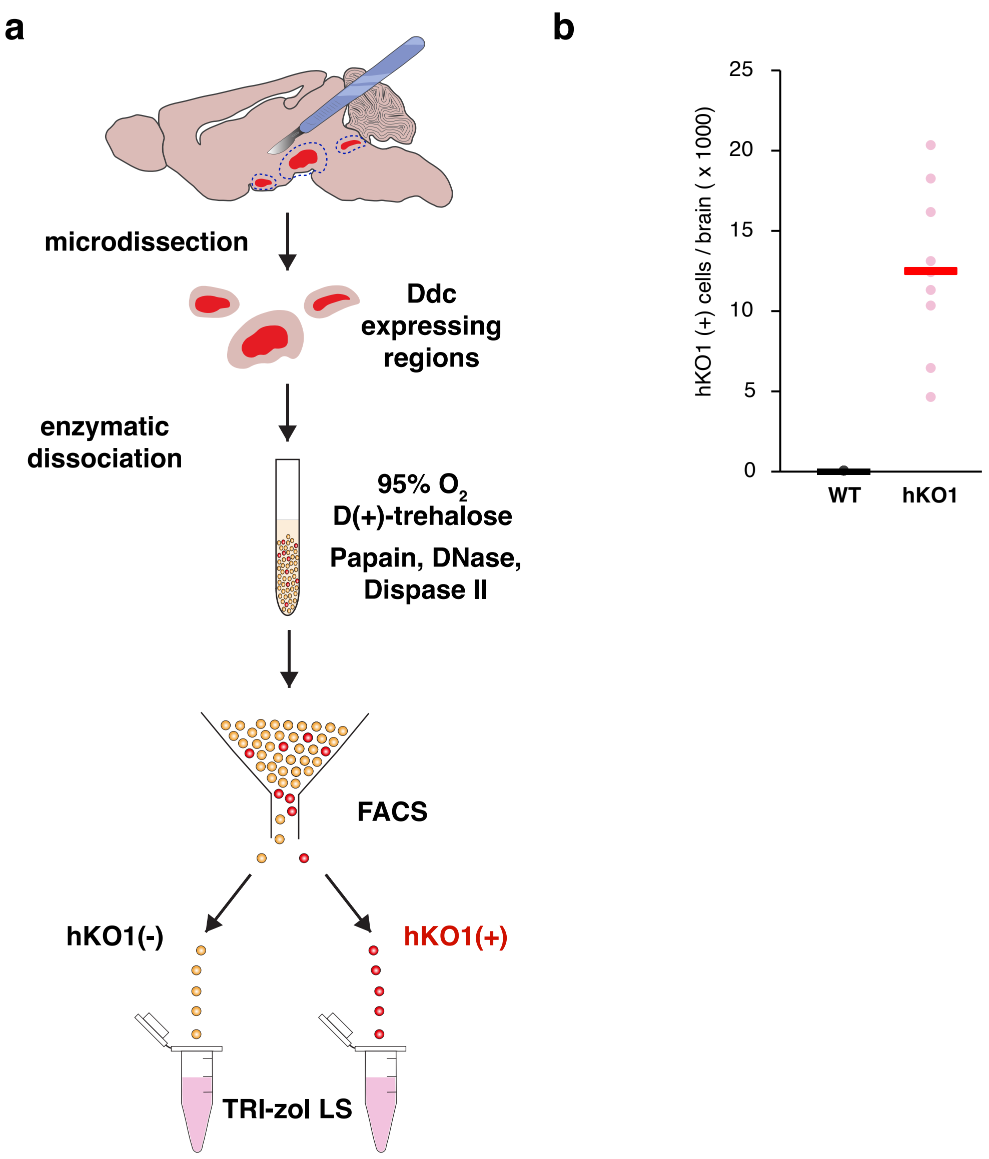

Supplementary Figure 2
Purification of the neurons from Ddc-hKO1 homozygous mouse brain using the flow cytometry.
(a) Schematic diagram showing the procedure of FACS-based purification of hKO1-positive cells from all *Ddc*-expressing regions of Ddc-hKO1 homozygous mice. **(b)** Number of hKO1-positive cells recovered from a single Ddc-hKO1 homozygous mouse brain. Each bar represents the average, and dots represent the actual number harvested from an individual brain.

Supplementary Figure 3

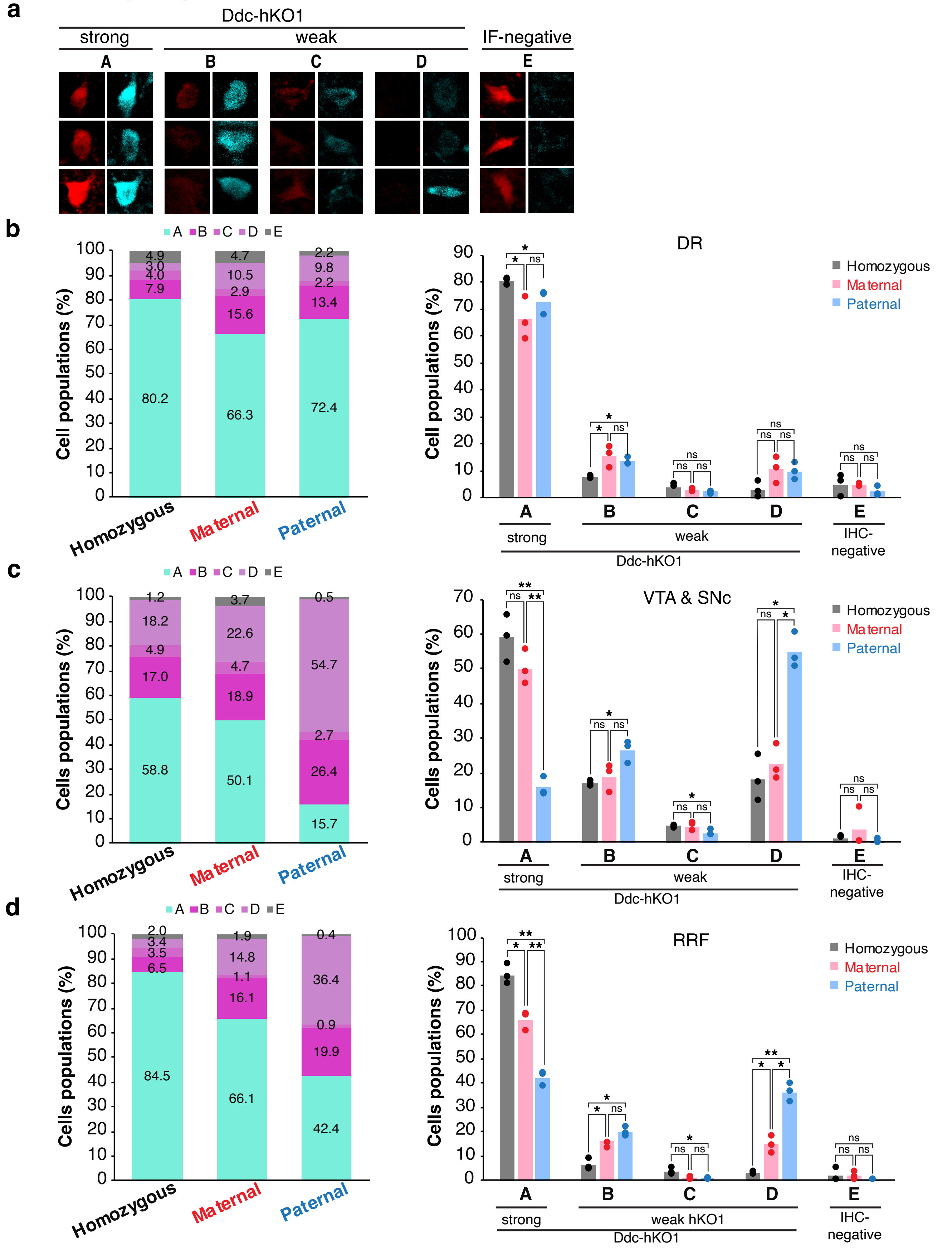

Supplementary Figure 3

Allelic expression of *Ddc* in mouse brain.

(a) Representative images of DDC immunofluorescence (DDC-IF) and Ddc-hKO1 combinations used in categorizing *Ddc* allelic expression pattern for cell counts. **(b-d)** Compound bar graphs on the left panel showing the total cell populations of each allelic expression pattern in brain regions of DR **(b)**, VTA & SNc **(c)** and RRF **(d)** from Ddc-hKO1 homozygous mice, maternally and paternally derived Ddc-hKO1 heterozygous mice. Numbers inside the bar indicating the total percentage of cell populations corresponding to the allelic expression pattern. Bar graphs on the right panel showing the average of total percentage of cell numbers of biological replicates in respective allelic expression category. Each dot represents the actual number. n = 3. *, P < 0.05; ** P < 0.01, student t-test. DR, dorsal raphe nucleus; VTA, ventral tegmented area; SNc, substantia nigra pars compacta; RRF, retrorubral field.
